# Efficacy of an intervention to reduce stigma towards people with skin diseases among health and body care professionals – a randomized controlled trial

**DOI:** 10.1111/ddg.16017

**Published:** 2026-01-16

**Authors:** Juliane Traxler, Catharina C. Braren‐von Stülpnagel, Matthias Augustin, Marius Grosser, Dennis Roggenkamp, Rachel Sommer

**Affiliations:** ^1^ German Center for Health Services Research in Dermatology (CVderm) Institute for Health Services Research in Dermatology and Nursing (IVDP) University Medical Center Hamburg‐Eppendorf (UKE) Hamburg Germany; ^2^ German Psoriasis Association (Deutscher Psoriasis Bund e.V.) Hamburg Germany; ^3^ Beiersdorf AG Hamburg Germany

**Keywords:** Intervention, psoriasis, RCT, skin diseases, stigmatization

## Abstract

**Background and Objectives:**

People with skin disease often experience stigmatization in health and body care settings, significantly impacting their quality of life. This parallel‐group randomized controlled trial evaluated a face‐to‐face group seminar aimed at reducing stigma towards people with skin disease among health and body care professionals (HBCPs).

**Patients and Methods:**

Cosmetologists, hairdressers, nurses, and physical therapists were randomized into an intervention (IG; n = 64) or control group (CG; n = 65). The IG received a seminar consisting of self‐awareness exercises, education and a patient encounter; the CG followed a seminar on “health at work”. Stereotype agreement, disease‐related misconceptions, desire for social distance, and behavioral intentions were assessed at baseline (t0), post‐intervention (t1), and 3 months follow‐up (t2).

**Results:**

The intervention group showed significant reductions over time in disease‐related misconceptions (t0–t1: 0.398, p < 0.001; t0–t2: 0.225, p < 0.001; ηp^2^ = 0.12) and in stereotype endorsement (t0–t1: 0.392, p < 0.001; t0–t2: 0.299, p = 0.002; ηp^2^ = 0.12). In both groups, the desire for social distance decreased immediately after the seminar (t0–t1_Intervention_: 0.186, p < 0.001; t0–t1_Control_: 0.135, p = 0.012) but returned to baseline at follow‐up (t0–t2_Intervention_: 0.097, p = 0.35; t0–t2_Control_: 0.016, p = 1.00).

**Conclusions:**

The seminar improved skin disease‐related stigmatizing beliefs and attitudes of HBCPs. Its integration into vocational training curricula or delivery in workshops to increase knowledge about skin diseases and to reduce prejudices in various professional groups is promising.

## INTRODUCTION

People with visible chronic skin diseases frequently face stigmatization. Several surveys showed that the majority of people with psoriasis reported having been discriminated against or humiliated,^1^, ^2^, ^3^ consistent with findings that others avoid physical contact,^4^ and that the disease hinders relationships.^5^ Similarly, people with other skin diseases including atopic dermatitis, hidradenitis suppurativa and others, also report having been met with disgusted looks (20.6%), experiencing refusal of leisure facilities (11.6%), and social rejection (15.6%).^6^ Studies from the US and Germany assessing attitudes towards people with chronic skin diseases among the general population corroborate this picture.^7^, ^8^, ^9^ For persons with chronic skin diseases, these experiences negatively affect psychological well‐being, social interactions, and quality of life.^1^, ^10^, ^11^, ^12^, ^13^

This significant impact on patients underlines the need to tackle stigma in the public. In a systematic review, Topp et al.^14^ identified only nine studies, of rather poor quality, that tested approaches aimed at reducing public stigma, all of which were targeted at leprosy. Hence, there is an urgent need for the development and thorough testing of approaches reducing stigma related to other skin diseases as well. In its 2014 resolution, the *World Health Assembly* (WHA) called on its member states to take action to increase acceptance of people with psoriasis, specifically.^15^ In response, the project ECHT,^16^ was conducted in Germany aiming to destigmatize chronic skin diseases among medical students,^17^ and educators in training.^18^ Participants took part in a seminar consisting of self‐reflection exercises, a theoretical lecture on skin diseases and stigma, and an encounter with a patient. This intervention structure is in line with Heijnders and van der Meij's,^19^ recommendation to combine education with a contact approach when addressing stigma at community level and indeed proved successful in reducing stigmatizing beliefs and attitudes towards persons with chronic skin diseases.^17^, ^18^

Another context in which persons with chronic skin diseases experience stigmatization is health and body care: nearly 10% of respondents in the Clear About Psoriasis Survey^1^ have been denied haircuts or cosmetic treatments. Similar numbers were observed 25 years ago.^20^ Hairdressers, cosmetologists, physical therapists and nurses have physical contact with their clients, and both their overt rejection and more subtle hesitations and facial expressions in reaction to skin diseases can have a substantial impact on patients’ well‐being and their willingness to use these services. Hence, this project aimed to adapt the “ECHT” intervention for body care professions and evaluate its effectiveness. It was hypothesized that the intervention group would show a larger reduction in stigmatizing attitudes towards people with chronic skin diseases compared to the control group.

## MATERIALS AND METHODS

### Participants

Participants were recruited through mailing lists and newsletters of vocational schools, professional associations and the local University Medical Centre, social media posts, and advertising at local hairdressers and beauty salons. Based on an *a priori* sample size calculation using G*Power,^21^ (F‐tests for repeated measures ANOVA, within‐between interaction) with the effect size obtained by Weinberger et al.,^18^ (interaction effect for behavioral intentions: η_p_^2^ = 0.02), 82 participants are needed to obtain a power of .80 (α = .05). To account for multiple testing and a potential dropout rate of up to 20% at follow‐up, we aimed to include 120 participants in total.

Self‐reported eligibility criteria comprised: being 18 years or older; working as a hairdresser, cosmetologist, nurse or physical therapist or being at least in the second year of training for one of these professions; being able to consent autonomously and to speak and understand German.

Participants provided written informed consent at the beginning of the seminar and received monetary compensation for taking part in the seminar (40 €) and filling in the follow‐up questionnaire (10 €). The study was carried out in compliance with the Helsinki Declaration and approved by the ethics committee of the local University Medical Centre (approval reference number: LPEK‐0395).

### Procedure

Participants signed up for one of the seminar dates on the study website. For reasons of feasibility, a randomization was applied where seminar dates were randomly assigned to be either intervention or control seminars.

Participants took part in an in‐person group seminar at the local University Medical Centre. Upon arrival, participants read the participant information and signed the informed consent form. Next, they filled in the pre‐test questionnaire (t0), followed by a brief introduction round. Subsequently, the intervention or control program (both 2.5 hours in length; for details, see below) was delivered by one of the authors (RS, JT). After completion, participants filled in the post‐test questionnaire (t1). Three months later, participants received the link to the follow‐up questionnaire (t2) via e‐mail.

*Intervention group*: In the intervention group, participants received a modified version of the “ECHT” seminar,^17^, ^18^ consisting of three components: *(1)* three self‐reflection exercises about participants’ own vulnerability to stigmatization, stigmatization risk in 24 different dermatological diseases, and participants’ reflection on which skin disease they could imagine living with the best and the least (30 min); *(2)* a lecture on skin diseases and stigma with case examples on urticaria and psoriasis (20 min); and *(3)* an encounter with a person with psoriasis who shared their personal experience living with a chronic skin disease with an emphasis on stigmatization and who answered questions from participants (60 min). Apart from the patient encounter, seminar contents were skin generic.

*Control group*: The control group received a seminar that was comparable to that of the intervention group in both structure and degree of required engagement/activity but focused on the participants’ health in the workplace consisting of: (*1*) a self‐reflection phase, in which participants reflected on their own experiences with physical and psychological stress at work (30 min); (*2*) a lecture focused on stress and stress management techniques (20 min); and (*3*) an e‐learning tool on mental health for employees (60 min).

### Outcome Measures

The following outcomes were assessed by means of self‐report via the platform Unipark (http://www.unipark.com) or using pen and paper.

#### Primary outcome measures

Instruments to assess stigmatization were adapted and developed by Pearl et al.^9^ and translated into German by Sommer et al.^17^ These instruments focused on psoriasis as a specific example.

*Stereotype endorsement*: Agreement with negative stereotypes about people with psoriasis was assessed using a scale consisting of eleven adjective pairs (e.g., unattractive – attractive). Participants were asked to mark the circle closest to the adjective they considered to describe a person with psoriasis (range: 1–5). Scores were averaged, with higher scores reflecting stronger endorsement of negative stereotypes. Internal consistency in this study was good at all time points (all *α≥*.83).

*Disease‐related misconceptions*: To measure agreement with common misconceptions about psoriasis, participants indicated their degree of agreement with 15 statements (e.g., “Psoriasis is contagious.”) on a 5‐point Likert scale ranging from “strongly disagree” to “strongly agree”. Scores were averaged, higher scores reflected stronger endorsement of these misconceptions. Internal consistency in this study was acceptable at all time points (all *α≥*.65).

*Desire for social distance*: Participants rated their desire for social distance towards persons with psoriasis in nine situations (e.g., shaking hands) on a 5‐point Likert scale ranging from “definitely not” to “definitely”. Scores were averaged, higher scores reflected a higher desire for distance. Internal consistency in this study was good at all time points (all *α≥*.86).

*Reported and intended behavior*: The Reported and Intended Behavior Scale, developed by Evans‐Lacko et al.,^22^ consists of four items assessing the presence of behavior (yes/no) in each of four contexts (living together with, working with, living nearby, and continuing a relationship with someone affected by a chronic skin disease) and four more items assessing intended behavior in these contexts in the future, rated on a 5‐point Likert scale ranging from “strongly disagree” to “strongly agree”. A sum score across the four “future behavior” items was calculated, with lower scores indicating more stigmatizing behavior. Internal consistency in this study was good at all time points (all *α≥*.81).

#### Secondary outcome measures

*Satisfaction with the seminar*: Participants’ satisfaction with the seminar was assessed at t1 using twelve items addressing general satisfaction with the seminar and its scope, personal and occupational relevance, preparedness for similar situations at work, and whether they would recommend the seminar to a colleague.^17^, ^18^ The statements were rated on a 5‐point Likert scale, ranging from “strongly agree” to “strongly disagree”, with lower scores indicating stronger satisfaction. Further secondary outcome measures are described in the Supplementary Material (S1).

### Statistical analyses

Statistical analyses were performed by a blinded analyst (CBvS) using Statistical Package for the Social Sciences (SPSS v. 27; IBM Corp., Armonk, NY, USA). Descriptive statistics were obtained for sociodemographic data, personal affliction with a skin disease, work experience and frequency of contact with people affected. Between‐group differences were analyzed using independent samples t‐tests, Mann‐Whitney U tests, and χ^2^ tests. Assumptions of normality and sphericity were checked by means of Shapiro‐Wilk and Mauchly's Test of Sphericity, respectively.

Intervention effects on the primary outcomes were analyzed by means of 2 (Group: intervention, control) x 3 (Time: t0, t1, t2) repeated‐measures analyses of variance (ANOVA). Subgroup analyses were performed to assess potential differences between the professions. Due to low sample sizes among hairdressers and cosmetologists but comparable vocational training, these professions were pooled into a *Beauty* group. Intervention effects on the primary outcomes by profession were analyzed through 2 (Group: intervention, control) x 3 (Time: t0, t1, t2) x 3 (Profession: beauty, nurses, physical therapists) repeated‐measures ANOVAs. For the variable *desire for social distance*, the assumption of sphericity was violated (p* = *0.001) and therefore Huynh‐Feldt corrected results are reported. P values were obtained from two‐tailed tests, with p < 0.05 indicating statistical significance. Bonferroni correction for multiple testing was applied. Effect sizes are reported as η_p_^2^, with η_p_^2^<0.06 indicating a small effect, η_p_^2^≥0.06 indicating a medium effect and η_p_^2^≥0.14 indicating a large effect ^23^. Significant effects were examined more closely using post‐hoc pairwise comparison.

### Ethics

The study was reviewed and approved by the local ethics committee of the University Medical Center Hamburg‐Eppendorf (LPEK‐0395). All participants provided written consent for the use and publication of their anonymized study data.

## RESULTS

### Sample Characteristics

**FIGURE 1:**
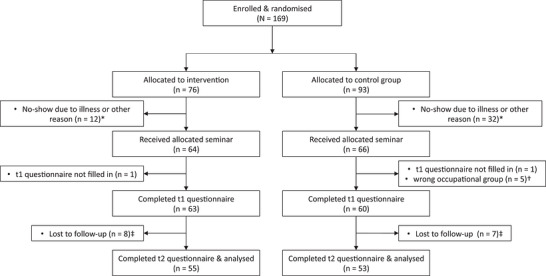
Participant flow chart. *At this stage, participants were unaware of their allocated group. ^†^These participants disclosed during the seminar that they did not belong to one of the target professions. ^‡^These participants did not respond to the e‐mail invitation nor the reminder to fill in the t2 questionnaire. The reasons for non‐response could not be identified.

Fifteen seminars were conducted between October 2022 and February 2023, with seven for the intervention group and eight for the control group to achieve roughly equal numbers of participants in both groups. All started at 6 or 6:30 p.m. on a weekday (Monday–Thursday). In total, 129 participants took part in these seminars, with 64 and 65 participants in the intervention and control groups, respectively. On average, 8.6 participants attended each seminar. Complete datasets for all three time points were available from 108 participants (t2 response rate: 90%), with 55 in the intervention group and 53 in the control group (Figure 1). Demographics of these participants are presented in Table 1. The two groups were comparable on all demographic variables and baseline scores of the primary outcome measures. The majority of participants (63%) were blind to their group membership, with no difference between the two groups. However, only 55.6% were blind to the study goal, with significantly more people blinded in the control group (69.8%) compared to the intervention group (41.8%; p *< *0.001).

**TABLE 1:** Descriptive statistics of sociodemographic data and baseline scores on the primary outcomes.

		Total (n = 108)	Intervention (n = 55)	Control (n = 53)
Gender	Female	95 (88%)	49 (89.1%)	46 (86.8%)
	Male	12 (11.1%)	5 (9.1%)	7 (13.2%)
	Diverse	1 (0.9%)	1 (1.8%)	–
Age, in years		32.12 ± 12.88	30.53 ± 12.62	33.67 ± 13.08
Occupation	Beauty	18 (16.6%)	10 (18.2%)	8 (15.1%)
	Nurse	41 (38.0%)	22 (40.0%)	19 (35.8%)
	PT	49 (45.4%)	23 (41.8%)	26 (49.1%)
Work experience, in years		9.04 ± 11.19	7.86 ± 10.54	10.28 ± 11.82
Affected by skin disease		18 (16.7%)	10 (18.2%)	8 (15.1%)
Frequency of contact with persons affected by a skin disease	Never	4 (3.7%)	3 (5.5%)	1 (1.9%)
	Rarely	30 (27.8%)	13 (23.6%)	17 (32.1%)
	Sometimes	38 (35.2%)	22 (40.0%)	16 (30.2%)
	Often	26 (24.1%)	11 (20.0%)	15 (28.3%)
	Daily	7 (6.5%)	4 (7.3%)	3 (5.7%)
	Missing	2 (1.9%)	1 (1.8%)	1 (1.9%)
Blind to study goal (%)		60 (55.6%)	23 (41.8%)	37 (69.8%)
Group membership	Correct	38 (35.2%)	18 (32.7%)	20 (37.7%)
	Incorrect	68 (63.0%)	36 (65.5%)	32 (60.4%)
	Unsure	2 (1.9%)	1 (1.8%)	1 (1.9%)
Stereotype endorsement		2.75 ± 0.62	2.73 ± 0.63	2.77 ± 0.61
Desire for social distance		1.67 ± 0.54	1.60 ± 0.49	1.75 ± 0.58
Disease‐related misconceptions		2.06 ± 0.43	2.08 ± 0.41	2.07 ± 0.45
Stigmatizing behavior		18.22 ± 2.50	18.43 ± 2.32	17.98 ± 2.68

*Note*: Beauty: hairdressers and cosmetologists, PT: physical therapists

### Primary Outcomes

**FIGURE 2:**
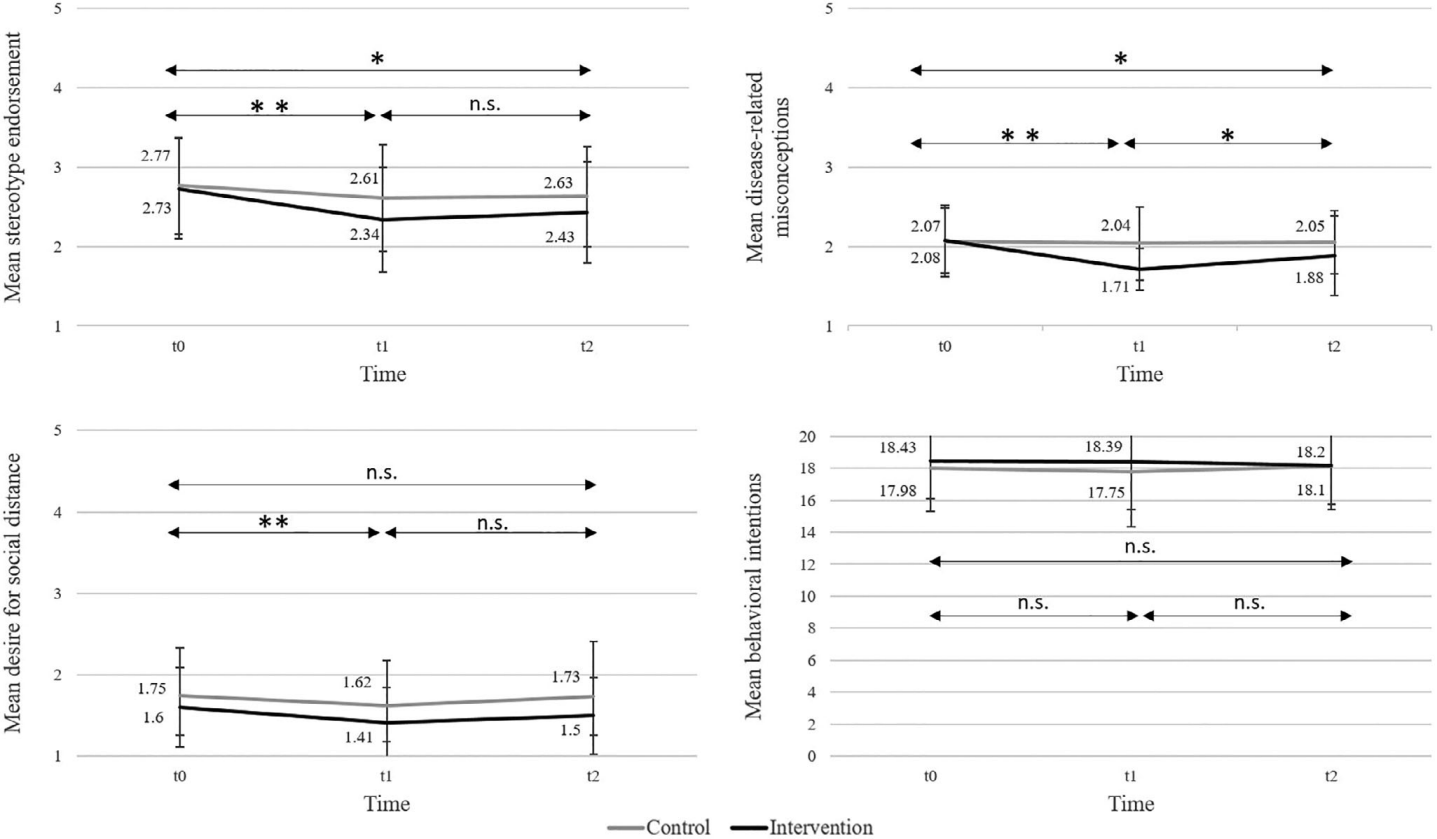
Mean values of the primary outcomes for intervention and control group at pre‐test (t0), post‐test (t1), and 3‐months follow‐up (t2). n.s., not significant. *p < 0.05; *p < 0.001.

Figure 2 shows the changes in the primary outcomes across time for the intervention and control group. Table 2 presents the means and standard deviations of primary outcomes at t0, t1 and t2.

**TABLE 2:** Descriptive statistics (mean, SD) of primary outcomes at pre‐test (t0), post‐test (t1) and follow‐up (t2) and changes over time by group.

	Intervention (n = 55)			Control (n = 53)		
	*Mean (SD)*	*Comparison*	*Difference*	*Mean (SD)*	*Comparison*	*Difference*
***Stereotype endorsement***						
t0	2.73 (0.63)	t1–t0	p < 0.001	2.77 (0.61)	t1–t0	p = 0.10
t1	2.34 (0.66)	t2–t0	p = 0.002	2.61 (0.67)	t2–t0	p = 0.33
t2	2.43 (0.64)	t2–t1	p = 0.69	2.63 (0.63)	t2–t1	p > 0.99
***Desire for social distance***						
t0	1.60 (0.49)	t1–t0	p < 0.001	1.75 (0.58)	t1–t0	p = 0.013
t1	1.41 (0.44)	t2–t0	p = 0.10	1.62 (0.56)	t2–t0	p = 0.81
t2	1.50 (0.47)	t2–t1	p = 0.16	1.74 (0.68)	t2–t1	p = 0.033
***Disease‐related misconceptions***						
t0	2.08 (0.41)	t1–t0	p < 0.001	2.07 (0.45)	t1–t0	p > 0.99
t1	1.71 (0.26)	t2–t0	p < 0.001	2.04 (0.46)	t2–t0	p > 0.99
t2	1.88 (0.50)	t2–t1	p = 0.003	2.05 (0.40)	t2–t1	p > 0.99
***Behavioral intentions***						
t0	18.43 (2.68)	t1–t0	p > 0.99	17.98 (2.32)	t1–t0	p > 0.99
t1	18.39 (3.42)	t2–t0	p > 0.99	17.75 (2.99)	t2–t0	p > 0.99
t2	18.20 (2.71)	t2–t1	p > 0.99	18.10 (2.71)	t2–t1	p = 0.97

*Stereotype endorsement*: A significant main effect of Time on stereotype endorsement was revealed, *F*(2,212) = 13.74, p* < *0.001, η_p_^2^ = 0.12. No main effect of Group, *F*(1,106) = 2.65, p* = *0.22, nor an interaction effect, *F*(2,212) = 2.30, p* = *0.10, were found. Post‐hoc tests indicated that in the intervention group, stereotype endorsement significantly decreased from t0 to t1 (difference_t0‐t1_: 0.392, p* < *0.001, 95% CI 0.22–0.57) and t2 (difference_t0‐t2_: 0.299, p* = *0.002, 95% CI 0.1–0.5) but remained stable between t1 and t2 (difference_t1‐t2_: ‐0.093, p* = *0.69, 95% CI –0.28–0.09). There was no significant difference between the three time points in the control group.

*Disease‐related misconceptions*: Significant main effects of Time, *F*(2,210) = 20.50, p* < *0.001, η_p_^2^ = 0.16, and Group, *F*(1,105) = 4.52, p* = *0.036, η_p_^2^ = 0.04, on disease‐related misconceptions were revealed as well as a significant interaction effect, *F*(2,210) = 14.81, p* < *0.001, η_p_^2^ = 0.12. Post‐hoc tests indicated that in the intervention group, disease‐related misconceptions decreased significantly from t0 to t1 (difference_t0‐t1_: 0.398, p* < *0.001, 95% CI 0.28–0.51) and t2 (difference_t0‐t2_: 0.225, p* < *0.001, 95% CI 0.12–0.33) but also increased significantly from t1 to t2 (difference_t1‐t2_: ‐0.173, p* = *0.003, 95% CI ‐0.30–(‐0.05)). There were no significant differences between any of the three time points in the control group.

*Desire for social distance*: Regarding desire for social distance towards people with psoriasis, significant main effects of Time, Huynh‐Feldt *F*(1.83,194.26) = 8.53, p* < *0.001, η_p_^2^ = 0.074, and Group, *F*(1,106) = 4.41, p* = *0.038, η_p_^2^ = 0.040, were found. The interaction effect, Huynh‐Feldt *F*(1.83,194.26) = 0.544, p* = *0.57, was not significant. Post‐hoc tests indicated that desire for social distance significantly decreased from t0 to t1 in both the intervention group (difference_t0‐t1_: 0.186, p* < *0.001, 95% CI 0.08–0.29) and the control group (difference_t0‐t1_: 0.135, p* = *0.012, 95% CI 0.02–0.25). However, values almost returned to baseline at T2 in both groups (Intervention group: difference_t0‐t2_: 0.097, p* = *0.35, 95% CI –0.05–0.25; Control group: difference_t0‐t2_: 0.016, p >0.99, 95% CI –0.136–0.168).

*Reported and intended behavior*. No significant main effect of Time, *F*(2,208) = 0.18, p* = *0.84, or Group, *F*(1,104) = 0.70, p* = *0.41, on behavioral intentions was found, nor was the interaction term significant, *F*(2,208) = 0.72, p* = *0.49.

### Secondary Outcomes

Satisfaction with the seminar and its perceived relevance were generally high. However, compared to the control group, participants in the intervention group were significantly more satisfied with the seminar overall (control: M = 4.45, SD = 0.70; intervention: M = 4.78, SD = 0.69), t(105.72) = –2.48, p = 0.015, felt better prepared for practice (control: M = 3.79, SD = 0.72; intervention: M = 4.29, SD = 1.12), t(92.49) = –2.77, p = 0.007, and were more willing to recommend the seminar to their colleagues (control: M = 4.60, SD = 0.60; intervention: M = 4.84, SD = 0.46), t(97.74) = –2.25, p = 0.027.

Results of subgroup analyses by profession, analyses controlling for prior experience with skin diseases, and data on port‐wine stain and on an additional secondary outcome (emotional responses to images of skin diseases) can be found in the online supplementary material (S2–S5).

## DISCUSSION

Based on the WHA's call to fight stigma,^15^ and the ECHT project,^17^, ^18^ this study evaluated the effectiveness of a group seminar in reducing stigmatizing attitudes and improving behavioral intentions towards chronic skin diseases in health and body care professionals. The results confirmed a significant decrease in stigmatizing attitudes, which can be partially attributed to the seminar: specifically, the intervention group held significantly fewer disease‐related misconceptions after attending the seminar compared to the control group. The intervention group also showed significantly reduced stereotype endorsement both at post‐test and 3 months follow‐up, although these changes were not significantly different compared to the control group. Surprisingly, social distance decreased significantly in both groups from baseline to post‐test, but these effects disappeared at follow‐up. Importantly, disease‐related misconceptions but not stereotype endorsement or desire for social distance towards people with port‐wine stain also decreased over time in the intervention group (online supplementary material S4). This indicates that the effects of the seminar generalize to other skin conditions that were only dealt with to a limited extent in the lecture or when meeting patients. No changes were observed for either group regarding behavioral intentions towards persons with chronic skin diseases. The seminar appeared equally suitable for the different professions (online supplementary material S2).

Results largely align with previous studies: both medical students and future educators in the ECHT study and people from the general population who took part in a stigma‐reduction program on Instagram^24^ reported fewer disease‐related misconceptions and less agreement with negative stereotypes after the intervention. However, only ECHT participants showed small changes in behavioral intentions, which did not persist over 3 months. This could have several reasons: from the start, self‐reported behavioral intentions towards persons with psoriasis were highly positive particularly in the current sample, such that a ceiling effect might have occurred. Moreover, while knowledge is considered a prerequisite for attitude and behavior change,^10^, ^25^, ^26^ and was shown to mediate the effects of contact with members of a stigmatized group on prejudicial attitudes,^27^ other factors like motivation play a role as well.^25^ The ECHT seminars were administered in the context of the participants’ studies and vocational training, which, despite their voluntary character, might have motivated participants to engage and learn even more. Furthermore, in contrast to the control group in the ECHT project, which comprised reading dermatology‐related scientific articles, the control group in this study received a seminar that resembled that of the intervention group in terms of structure, duration, and participation levels, which may have contributed to the smaller effects on behavioral intentions.

Regarding generalizability of the present findings, the majority of the sample consisted of women (88%), which is slightly higher than what would be expected based on the gender representation in the target professions.^28^, ^29^, ^30^ However, the participants’ ages spanned the range of the regular working population (20–66 years) and they came from diverse socioeconomic backgrounds (59.3% of the sample reported a monthly household income of 2,000 € or less, 14% reported one of > 4,000 €; 60% had a high school diploma (*Fach‐)Abitur*, 7% held a university degree). Hence, our results are generalizable to the wider target population.

### Limitations and future directions

Several limitations need to be taken into account when interpreting the results of this study: first, blinding of participants was not entirely successful. Although almost two thirds of participants were blind to their group membership, only half of the sample was blind to the study goal. This may be due to the strong focus of the questionnaires on attitudes towards skin diseases and may have encouraged socially desirable responses. Furthermore, many participants were classmates or co‐workers and, despite instructions not to do so, may have shared their experiences with each other, so that participants learned about the content of the other seminar as well. Another limitation is the relatively short follow‐up period of three months.

Future studies should test longer‐term effects, use behavioral measures to circumvent the problem of social desirability, and assess attitudes towards other skin diseases. In addition, identifying the active elements of the seminar to potentially shorten it, exploring the effectiveness of an online version of the seminar, and testing the utility of a booster session could help to improve outcomes while making it more feasible for integration in vocational training curricula.

## CONCLUSIONS

The seminar was successful in reducing stigmatizing misconceptions about chronic skin diseases but not stigmatizing behaviors among health and body care professionals. The results highlight the need for further improvement of the intervention but also warrant implementation in vocational training curricula. A broader dissemination of this training may ultimately contribute to tackling the widespread stigma associated with dermatological diseases and improving well‐being, social participation and quality of life of the persons affected.

## FUNDING STATEMENT

This study was funded by Eucerin, Beiersdorf AG. The funder had no role in the study design, data collection, data analysis, interpretation of the data, in the writing of the report, or in the decision to submit the paper for publication.

## CONFLICT OF INTEREST STATEMENT

M.A. has served as consultant and paid speaker and has received research grants, honoraria and travel expense reimbursements for consulting and scientific lectures and has participated in clinical trials sponsored by AbbVie, Almirall, Amgen, Beiersdorf AG, Biogen, BMS, Boehringer Ingelheim, Celgene, Centocor, Eli Lilly, Galderma, Janssen‐Cilag, Leo Pharma, medac, MSD, Novartis, Pfizer and Sandoz. D.R. is currently employed by Beiersdorf AG. J.T., C.B.v.S., M.G. and R.S. declare no conflict of interest.

## Supporting information



Supplementary information

## Data Availability

The study protocol, intervention materials, and analysis code can be found under: https://osf.io/zaqg6. Individual participant data that underlie the results reported in this article, after de‐identification (text, tables, figures, and appendices), as well as the statistical analysis plan and analytic code are available from the corresponding author upon reasonable request, beginning 3 months and ending 5 years following article publication. Data can be shared with investigators whose proposed use of the data has been approved by an independent review committee (learned intermediary) identified for this purpose.
