# Supplementary material for: Efficacy of an intervention to reduce stigma towards people with skin diseases among health and body care professionals – a randomized controlled trial
Source: J Dtsch Dermatol Ges. 2026 Jan 16;24(8):1077–85. doi: 10.1111/ddg.16017 (PMC13435086; doi:10.1111/ddg.16017)
Supplement: Supplementary file 1 [file DDG-24-1077-s001.docx]

**Reducing Stigma towards People with Skin Diseases among Health and Body Care Professionals: an RCT**

**S1. Secondary Analyses**

*Blinding to study goal and group membership.* At follow-up, participants were asked to describe what they thought was the goal of the study and to indicate whether they believed they were in the intervention group, the control group, or did not know which group they belonged to.

*Controlling for Confounders.* In order to control for prior experience with skin diseases, the four rmANOVAs on the effects of Group and Time on the primary outcome measures were repeated with the following covariates: being affected by a skin disease (yes/no), knowing someone else with a skin disease [yes/no; based on Reported and Intended Behavior Scale items 1–4^1^], and contact frequency to people with a skin disease (1=“never” to 5=“daily”).

*Port-Wine Stain.* As the intervention content was designed to be skin-generic, the questionnaires assessing stereotype endorsement, disease-related misconceptions and desire for social distance were administered a second time with a focus on port-wine stain to test whether potential changes in stigmatizing attitudes generalised to other skin conditions.

*Outcome Measure Emotional Responses.* A subscale of the Desire for Social Distance Scale^2^ assessed emotional responses to three images of persons with psoriasis and two images of persons with port-wine stain. Images were standardized with black background, and patients had consented to their images being used for research purposes. Participants were asked to rate the extent to which they felt six emotions (blame, compassion, contempt, curiosity, disgust, pity) on a scale from 1=“true” to 5=“false” in response to the psoriasis and port-wine stain images, respectively.

**S2. Subgroup Analyses**

Table S1 presents means and standard deviations of primary outcomes at t0, t1 and t2 by profession.

*Stereotype endorsement.* A significant main effect of Time on stereotype endorsement was found, *F*(2,204)=10.51, *p<*0.001, η_p_^2^=0.09. No main effect of Group nor any significant interaction effects were found. Post-hoc tests indicated that in the intervention group, stereotype endorsement decreased significantly from t0 to t1 (difference_t0-t1_: 0.538, *p<*0.001, 95% CI 0.27–0.81) and t2 (difference_t0-t2_: 0.494, *p<*0.001, 95% CI 0.18–0.81) but remained stable between t1 and t2 (difference_t1-t2_: -0.043, *p=*1.00, 95% CI -0.33–0.25) only among the physical therapists. There were no significant differences between the three time points in any of the other professions.

*Disease-related misconceptions.* A significant main effect of Time, *F*(2,202) = 25.13, *p<*0.001, η_p_^2^=0.20, as well as significant interaction effects between Time and Group, *F*(2,202)=15.11, *p<*0.001, η_p_^2^=0.13 and between Time and Profession, *F*(4,202)=3.93, *p=*0.004, η_p_^2^=0.07, were observed. The three-way interaction between Time, Group and Profession was not significant. Post-hoc tests indicated that in the intervention group, disease-related misconceptions decreased significantly from t0 to t1 in all professions (beauty: difference_t0-t1_: 0.687, *p<*0.001, 95% CI 0.43–0.95); physical therapists: difference_t0-t1_: 0.426, *p<*0.001, 95% CI 0.26–0.60); nurses: difference_t0-t1_: 0.229, *p=*0.007, 95% CI -0.05–0.41). Values were still significantly lower at t2 compared to t0 for both the beauty group and physical therapists (beauty: difference_t0-t2_: 0.267*, p=*0.018, 95% CI 0.04–0.50; physical therapists: difference_t0-t2_: 0.380, *p<*0.001, 95% CI 0.23–0.53) but not the nurses (difference_t0-t2_: 0.035, *p=*1.00, 95% CI -0.12–0.19). However, the beauty group showed a significant increase in disease-related misconceptions from t1 to t2 (difference_t1-t2_: -0.420, *p=*0.001, 95% CI -0.70–(-0.14)) while the other professions did not (physical therapists: difference_t1-t2_: -0.046, *p=*1.00, 95% CI -0.23–0.14; nurses: difference_t1-t2_: -0.194, *p=*0.051, 95% CI -0.39–0.00). There were no significant differences between any of the three time points in the control group.

*Desire for social distance.* For desire for social distance towards people with psoriasis, a significant main effect of Time, *Greenhouse-Geisser* *F*(1.77,180.557)=6.45, *p=*0.003, η_p_^2^=0.06, and a Time x Profession interaction effect, *Greenhouse-Geisser* *F*(3.54, 180.557)=2.91, *p=*0.028, η_p_^2^=0.05, were observed. Post-hoc tests indicated that in the control group, desire for social distance significantly decreased from t0 to t1 (difference_t0-t1_: 0.233, *p=*0.006, 95% CI 0.05–0.41) among the nurses. However, values increased significantly from t1 to t2 (difference_t1-t2_: -0.241, *p=*0.046, 95% CI -0.48–(-0.01)) and were no longer significantly different from t0 (difference_t0-t2_: -0.008, *p=*1.00, 95% CI -0.25–0.24). Moreover, desire for social distance significantly decreased from t0 to t1 (difference_t0-t1_: 0.154, *p=*0.048, 95% CI 0.01–0.31) and remained stable between t1 and t2 (difference_t1-t2_: -0.003, *p=*1.00, 95% CI -0.21–0.20) in the physical therapists group. In the intervention group, desire for social distance also significantly decreased from t0 to t1 (difference_t0-t1_: 0.276, *p<*0.001, 95% CI 0.11–0.44) and remained stable between t1 and t2 (difference_t1-t2_: -0.09, *p=*0.987, 95% CI -0.30–0.13) in the physical therapists group.

*Reported and intended behavior.* No significant main or interaction effects on behavioral intentions were found.

**Table S1.** Descriptive statistics (mean, SD) of primary outcomes at pre-test (t0), post-test (t1) and follow-up (t2) and changes over time by group and profession

| **Variable** | **Intervention (n=55)** | | | **Control (n=53)** | | |
| --- | --- | --- | --- | --- | --- | --- |
|  | **Mean (SD)** | **Comparison** | **Difference** | **Mean (SD)** | **Comparison** | **Difference** |
| **Beauty** | | |  |  |  |  |
| Stereotype endorsement | | |  |  |  |  |
| t0 | 2.82 (0.90) | t1–t0 | *p=*0.114 | 2.76 (0.78) | t1–t0 | *p=*1.000 |
| t1 | 2.46 (0.77) | t2–t0 | *p=*1.000 | 2.75 (1.02) | t2–t0 | *p=*0.708 |
| t2 | 2.65 (0.57) | t2–t1 | *p=*0.943 | 2.50 (0.79) | t2–t1 | *p=*0.649 |
| Desire for social distance | |  |  |  |  |  |
| t0 | 1.75 (0.70) | t1–t0 | *p=*0.153 | 1.72 (0.65) | t1–t0 | *p=*0.476 |
| t1 | 1.55 (0.60) | t2–t0 | *p=*1.000 | 1.88 (0.88) | t2–t0 | *p=*0.063 |
| t2 | 1.79 (0.44) | t2–t1 | *p=*0.248 | 2.09 (1.27) | t2–t1 | *p=*0.525 |
| Disease-related misconceptions | | |  |  |  |  |
| t0 | 2.48 (0.72) | t1–t0 | *p<*0.001 | 2.39 (0.52) | t1–t0 | *p=*0.633 |
| t1 | 1.79 (0.34) | t2–t0 | *p=*0.018 | 2.24 (0.53) | t2–t0 | *p=*0.259 |
| t2 | 2.21 (0.82) | t2–t1 | *p=*0.001 | 2.21 (0.49) | t2–t1 | *p=*1.000 |
| Behavioral intentions | |  |  |  |  |  |
| t0 | 17.00 (5.21) | t1–t0 | *p=*0.829 | 18.14 (2.55) | t1–t0 | *p=*0.986 |
| t1 | 16.20 (6.56) | t2–t0 | *p=*0.209 | 17.25 (3.06) | t2–t0 | *p=*0.827 |
| t2 | 15.80 (4.87) | t2–t1 | *p=*1.000 | 16.13 (4.19) | t2–t1 | *p=*1.000 |
| **Nurses** | | | | | | |
| Stereotype endorsement | | | | | | |
| t0 | 2.67 (0.63) | t1–t0 | *p=*0.079 | 2.77 (0.50) | t1–t0 | *p=*0.046 |
| t1 | 2.41 (0.70) | t2–t0 | *p=*0.750 | 2.47 (0.53) | t2–t0 | *p=*1.000 |
| t2 | 2.52 (0.71) | t2–t1 | *p=*1.000 | 2.66 (0.54) | t2–t1 | *p=*0.467 |
| Desire for social distance | | | | | | |
| t0 | 1.58 (0.53) | t1–t0 | *p=*0.657 | 1.77 (0.52) | t1–t0 | *p=*0.006 |
| t1 | 1.50 (0.49) | t2–t0 | *p=*1.000 | 1.53 (0.40) | t2–t0 | *p=*1.000 |
| t2 | 1.52 (0.57) | t2–t1 | *p=*1.000 | 1.77 (0.44) | t2–t1 | *p=*0.046 |
| Disease-related misconceptions | | | | | | |
| t0 | 1.95 (0.36) | t1–t0 | *p=*0.007 | 1.88 (0.34) | t1–t0 | *p=*1.000 |
| t1 | 1.72 (0.29) | t2–t0 | *p=*1.000 | 1.87 (0.39) | t2–t0 | *p=*1.000 |
| t2 | 1.91 (0.43) | t2–t1 | *p=*0.051 | 1.91 (0.31) | t2–t1 | *p=*1.000 |
| Behavioral intentions | | | | | | |
| t0 | 18.95 (1.73) | t1–t0 | *p=*1.000 | 18.21 (1.93) | t1–t0 | *p=*1.000 |
| t1 | 18.77 (2.39) | t2–t0 | *p=*1.000 | 18.53 (1.90) | t2–t0 | *p=*1.000 |
| t2 | 18.48 (1.63) | t2–t1 | *p=*1.000 | 18.42 (2.52) | t2–t1 | *p=*1.000 |
| **Physical therapists** | | | | | | |
| Stereotype endorsement | | | | | | |
| t0 | 2.75 (0.51) | t1–t0 | *p<*0.001 | 2.77 (0.65) | t1–t0 | *p=*1.000 |
| t1 | 2.22 (0.57) | t2–t0 | *p<*0.001 | 2.67 (0.64) | t2–t0 | *p=*1.000 |
| t2 | 2.26 (0.57) | t2–t1 | *p=*1.000 | 2.66 (0.68) | t2–t1 | *p=*1.000 |
| Desire for social distance | | | | | | |
| t0 | 1.55 (0.35) | t1–t0 | *p<*0.001 | 1.75 (0.62) | t1–t0 | *p=*0.048 |
| t1 | 1.27 (0.26) | t2–t0 | *p=*0.126 | 1.59 (0.53) | t2–t0 | *p=*0.252 |
| t2 | 1.36 (0.32) | t2–t1 | *p=*0.987 | 1.60 (0.56) | t2–t1 | *p=*1.000 |
| Disease-related misconceptions | | | | | | |
| t0 | 2.09 (0.33) | t1–t0 | *p<*0.001 | 2.12 (0.43) | t1–t0 | *p=*1.000 |
| t1 | 1.67 (0.19) | t2–t0 | *p<*0.001 | 2.11 (0.42) | t2–t0 | *p=*1.000 |
| t2 | 1.71 (0.26) | t2–t1 | *p=*1.000 | 2.10 (0.41) | t2–t1 | *p=*1.000 |
| Behavioral intentions | | | | | | |
| t0 | 18.61 (1.47) | t1–t0 | *p=*1.000 | 17.77 (2.58) | t1–t0 | *p=*0.933 |
| t1 | 19.04 (1.58) | t2–t0 | *p=*1.000 | 17.31 (3.52) | t2–t0 | *p=*1.000 |
| t2 | 19.00 (1.48) | t2–t1 | *p=*1.000 | 18.08 (2.34) | t2–t1 | *p=*0.383 |

**S3. Prior Experience with Skin Diseases**

*Being affected by a skin disease*. The rmANCOVAs on stereotype endorsement, desire for social distance and disease-related misconceptions referring to both psoriasis and port-wine stain, as well as on behavioral intentions all found non-significant effects of whether or not participants were affected by a skin disease (all *p*>0.06). The effects of Time and Group on the outcome variables were comparable across participants who reported having a skin disease themselves (n=18) and those who did not.

*Knowing someone else with a skin disease*. The rmANCOVAs on stereotype endorsement, desire for social distance, and behavioral intentions found no significant effects of knowing someone else with a skin disease (all *p*>0.08). Importantly, knowing someone else with a skin disease significantly predicted disease-related misconceptions concerning psoriasis (*p*=0.002) but not port-wine stain (*p*=0.513). The effect of Group on disease-related misconceptions concerning psoriasis remained significant after controlling for knowing someone else with a skin disease, F(1,104)=5.535, p=0.021.

*Contact frequency*. Higher contact frequency significantly predicted lower desire for social distance towards persons with psoriasis (*p*=0.024) and port-wine stain (*p*=0.029), fewer disease-related misconceptions about psoriasis (*p*<0.001), and more positive behavioral intentions towards persons with a skin disease (*p*<0.001). The effects of Group on desire for social distance towards persons with psoriasis, F(1,103)=4.121, p=0.045, and on disease-related misconceptions about psoriasis, F(1,102)=4.709, p=.032, both remained significant after controlling for contact frequency. The effects of Group on desire for social distance towards persons with port-wine stain, F(1,102)=1.282, p=0.260, and on behavioral intentions, F(1,102)=0.996, p=0.321, remained non-significant after controlling for contact frequency. The rmANCOVAs on stereotype endorsement (psoriasis, port-wine stain) and disease-related misconceptions about port-wine stain found no significant effects of contact frequency (all *p*>0.60).

**S4. Port-Wine Stain Analyses**

*Stereotype endorsement.* No main effects of Time, *Huynh-Feldt* *F*(1.84,195.09)=0.50, *p=*0.592, or Group on stereotype endorsement, *F*(1,106)=3.37, *p=*0.069, nor an interaction effect, *Huynh-Feldt* *F*(1.84,195.09)=1.21, *p=*0.297, were found. Furthermore, the subgroup analysis showed no interactions with profession indicating that results were comparable across the three professions.

*Disease-related misconceptions.* Main effects of Time, *F*(2,210)=2.652, *p=*0.073, and Group, *F*(1,105)=0.27, *p=*0.606, on disease-related misconceptions were not significant. However, a significant interaction effect was revealed, *F*(2,210)=4.845, *p=*0.009, η_p_^2^=0.04. Post-hoc tests indicated that in the intervention group, disease-related misconceptions decreased significantly from t0 to t2 (difference_t0-t2_: 0.241, *p=*0.003, 95% CI 0.07–0.41). The decrease from t0 to t1 did not reach significance (difference_t0-t1_: 0.199, *p=*0.052, 95% CI -0.001–0.40). There were no significant differences between any of the three time points in the control group. The subgroup analysis showed a significant interaction between Time and Group, *F*(2,202)=4.07, *p=*0.019, η_p_^2^=.04. No other main or interaction effects were found. Post-hoc tests showed a significant decrease in disease-related misconceptions among nurses in the intervention group between t0 and t2 (difference_t0-t2_: 0.298, *p=*0.029, 95% CI 0.02–0.57). No significant changes were observed in the other professions nor in the control group.

*Desire for social distance.* No main effects of Time, *Huynh-Feldt* *F*(1.68,176.50)=0.035, *p=*0.946, or Group, *F*(1,105)=1.996, *p=*0.161, on desire for social distance nor an interaction effect, *Huynh-Feldt* *F*(1.68,176.50)=1.583, *p=*0.211, were found. The subgroup analysis showed no interactions with profession indicating that results were comparable across the three professions.

**S5. Emotional Response Analyses**

*Emotional responses to psoriasis images.* A significant main effect of Time, *Greenhouse-Geisser* *F*(1.52,161.51)=8.65, *p<*0.001, on compassion was found, while the main effect of Group, *F*(1,106)=0.02, *p=*0.898, and the interaction effect were not significant, *Greenhouse-Geisser* *F*(2,210)=1.15, *p=*0.309. Post-hoc tests indicated that in the intervention group, compassion significantly decreased from t0 (difference_t0-t2_: 0.309, *p=*0.005, 95% CI 0.08–0.54) and t1 (difference_t1-t2_: 0.297, *p=*0.012, 95% CI 0.05–0.54) to t2 while there was no difference between t0 and t1 (difference_t0-t1_: 0.012, *p=*1.00, 95% CI -0.13–0.15). There were no significant differences between any of the three time points in the control group. Similarly, a significant main effect of Time, *Huynh-Feldt* *F*(1.81,191.99)=4.98, *p=*0.010, on pity was found, while the main effect of Group, *F*(1,106)=0.03, *p=*0.863, and the interaction effect were not significant, *Huynh-Feldt* *F*(1.81,191.99)=0.27, *p=*0.745. Post-hoc tests showed that in the control group, pity significantly decreased from t0 to t2 (difference_t0-t2_: 0.289, *p=*0.037, 95% CI 0.01–0.57) while there was no difference between t0 and t1 (difference_t0-t1_: 0.201, *p=*0.096, 95% CI -0.02–0.43) or between t1 and t2 (difference_t1-t2_: 0.088, *p=*1.00, 95% CI -0.23–0.40). There were no significant differences between any of the three time points in the intervention group. A significant main effect of Time, *Huynh-Feldt* *F*(1.86,196.76)=4.32, *p=*0.017, on blame was equally found, while the main effect of Group, *F*(1,106)=0.68, *p=*0.413, and the interaction effect were not significant, *Huynh-Feldt* *F*(1.86,196.76)=1.02, *p=*0.359. Post-hoc tests showed that in the intervention group, blame significantly decreased from t0 to t1 (difference_t0-t1_: 0.273, *p=*0.014, 95% CI 0.04–0.50) and significantly increased again from t1 to t2 (difference_t1-t2_: -0.333, *p=*0.026, 95% CI -0.63–(-0.03)), while there was no difference between t0 and t2 (difference_t0-t2_: -0.061, *p=*1.00, 95% CI -0.36–0.24). There were no significant differences between any of the three time points in the control group. Moreover, significant main effects of Time, *Huynh-Feldt* *F*(1.94,205.88)=30.66, *p<*0.001, and Group, *F*(1,106)=5.09, *p=*0.026, on disgust were found, while the interaction effect was not significant, *Huynh-Feldt* *F*(1.94,205.88)=2.02, *p=*0.136. Post-hoc tests showed that in the control group, disgust significantly decreased from t0 to t1 (difference_t0-t1_: 0.541, *p=*0.01, 95% CI 0.19–0.90) and significantly increased again from t1 to t2 (difference_t1-t2_: -0.321, *p=*0.023, 95% CI -0.61–(-0.03)), while there was no difference between t0 and t2 (difference_t0-t2_: 0.220, *p=*0.313, 95% CI -0.11–0.55). In the intervention group, disgust significantly decreased from t0 to t1 (difference_t0-t1_: 0.915, *p<*0.001, 95% CI 0.57–1.26) and significantly increased again from t1 to t2 (difference_t1-t2_: -0.539, *p<*0.001, 95% CI -0.82–(-0.26)). Yet, disgust levels at t2 were still significantly lower than at t0 (difference_t0-t2_: 0.376, *p=*0.016, 95% CI 0.06–0.70). The main effects of Time, *F*(2,212)=0.76, *p=*0.468, and Group, *F*(1,106)=0.98, *p=*0.324, on curiosity were not significant but a significant interaction effect was revealed, *F*(2,212)=5.19, *p=*0.006. Post-hoc tests showed that in the control group, curiosity significantly decreased from t0 to t2 (difference_t0-t2_: 0.409, *p=*0.020, 95% CI 0.05–0.77) while there was no difference between t0 and t1 (difference_t0-t1_: 0.270, *p=*0.105, 95% CI -0.04–0.58) or between t1 and t2 (difference_t1-t2_: 0.055, *p=*1.00, 95% CI -0.30–0.41). There were no significant differences between any of the three time points in the intervention group. Lastly, no main or interaction effects on contempt were found.

*Emotional responses to port-wine stain images.* A significant main effect of Time, *F*(2,210)=4.11, *p=*0.018, on compassion was found, while the main effect of Group, *F*(1,105)=0.20 *p=*0.658, and the interaction effect were not significant, *F*(2,210)=1.08, *p=*0.342. Post-hoc tests did not show any significant differences between the three time points for either group. A significant main effect of Time, *Huynh-Feldt* *F*(1.89,198.78)=5.66, *p=*0.005, on disgust was equally found, while the main effect of Group, *F*(1,105)=3.58, *p=*0.061, and the interaction effect were not significant, *Huynh-Feldt* *F*(1.89,198.78)=1.18, *p=*0.311. Post-hoc tests indicated that in the intervention group, experience of disgust significantly increased from t1 to t2 (difference_t1-t2_: -0.333, *p=*0.007, 95% CI -0.59–(-0.07)). However, disgust levels at t1 (difference_t0-t1_: 0.185, *p=*0.108, 95% CI -0.03–0.40) and t2 (difference_t0-t2_: -0.148, *p=*0.577, 95% CI -0.42–0.13) were not significantly different from t0. There were no significant differences between any of the three time points in the control group. No main or interaction effects on blame, contempt, curiosity, or pity were found.

**References**

1	Evans-Lacko S, Rose D, Little K et al. Development and psychometric properties of the reported and intended behaviour scale (RIBS): a stigma-related behaviour measure. Epidemiology and psychiatric sciences 2011; 20: 263–71.

2	Pearl RL, Wan MT, Takeshita J et al. Stigmatizing attitudes toward persons with psoriasis among laypersons and medical students. Journal of the American Academy of Dermatology 2019; 80: 1556–63.
